# Intracellular activation of EGFR by fatty acid synthase dependent palmitoylation

**DOI:** 10.18632/oncotarget.5252

**Published:** 2015-09-12

**Authors:** Lakshmi Reddy Bollu, Rajashekhara Reddy Katreddy, Alicia Marie Blessing, Nguyen Pham, Baohui Zheng, Xu Wu, Zhang Weihua

**Affiliations:** ^1^ Department of Biology and Biochemistry, College of Natural Sciences and Mathematics, University of Houston, Houston, Texas, USA; ^2^ Cutaneous Biology Research Center, Massachusetts General Hospital, Harvard Medical School, Charlestown, Massachusetts, USA

**Keywords:** EGFR, fatty acid synthase, palmitoylation, palmitoyl transferases, cancer

## Abstract

Epidermal growth factor receptor (EGFR) is an oncogenic receptor tyrosine kinase. Canonically, the tyrosine kinase activity of EGFR is regulated by its extracellular ligands. However, ligand-independent activation of EGFR exists in certain cancer cells, and the underlying mechanism remains to be defined. In this study, using PC3 and A549 cells as a model, we have found that, in the absence of extracellular ligands, a subpopulation of EGFR is constitutively active, which is needed for maintaining cell proliferation. Furthermore, we have found that fatty acid synthase (FASN)-dependent palmitoylation of EGFR is required for EGFR dimerization and kinase activation. Inhibition of FASN or palmitoyl acyltransferases reduced the activity and down-regulated the levels of EGFR, and sensitized cancer cells to EGFR tyrosine kinase inhibitors. It is concluded that EGFR can be activated intracellularly by FASN-dependent palmitoylation. This mechanism may serve as a new target for improving EGFR-based cancer therapy.

## INTRODUCTION

Epidermal growth factor receptor (EGFR) is a member of the epidermal growth factor receptor tyrosine kinase family (ErbB family) that includes four members, EGFR (HER1), HER2/Neu, HER3 and HER4. EGFR is frequently overexpressed in many human cancers and correlates with cancer progression, metastasis, and poor prognosis [1]. Structurally, EGFR is compartmentalized into three major domains; an extra cellular domain (ECD), a trans-membrane domain (TMD) and an intra-cellular domain (ICD). The ECD has a ligand binding domain (LBD); the TMD consists of 23 amino acids and anchors EGFR to the plasma membrane; and the ICD consists of the tyrosine kinase domain and a tyrosine rich domain that transduces signals to its downstream pathways such as the PI3K/Akt. Upon binding to its ligand, EGFR undergoes homo- or hetero-dimerization with another ErbB member. Dimerization of the receptor induces intermolecular auto-phosphorylation of EGFR which in turn initiates the activation of downstream signaling cascades. EGFR-targeted therapies have been developed based on the understanding of ligand-dependent and kinase-dependent functions of EGFR. Available anti-EGFR agents include EGFR monoclonal antibodies that prevent EGFR from binding to its extracellular ligands and small molecule tyrosine kinase inhibitors (TKIs) that block the tyrosine kinase domain in EGFR by competing with ATP [2]. However, interfering with EGFR signaling by these anti-EGFR reagents has produced limited effects at the clinic [3]. For example, inhibition of the tyrosine kinase of EGFR by TKIs has produced responding rates range between 10–20% in lung cancer patients; however, all patients acquired resistance within few months after treatment,[4, 5] and prostate cancer, a type of cancer where EGFR is commonly overexpressed, is innately resistant to EGFR TKIs [6, 7].

In cancer cells, EGFR and its downstream pathways can be constitutively activated independent of external signals [8, 9]. Overexpression of EGFR instantly leads to constitutive activation of EGFR in the absence of ligands [8]. The EGFR vIII, which lacks the ability of ligand binding, is overexpressed and constitutively active in cancer cells of glioblastoma multiforme [10]. Similarly, enhanced EGFR constitutive activation was observed in EGFR mutants with amino acid substitutions in the tyrosine kinase domain [11, 12]. The ligand-independent activation of EGFR suggests that EGFR can be activated intracellularly. In support of this possibility, studies have shown that EGFR can be dimerized independent of ligands, [13] and EGFR can be activated by fatty acid synthase (FASN) [14]. However, the intracellular mechanism by which EGFR is dimerized and activated independent of extracellular ligand remains to be defined. FASN-dependent protein palmitoylation regulates protein functions [15–19]. Previously, we have reported that EGFR physically interacts with FASN,[15] which led us to hypothesize that *de novo* synthesized palmitate by FASN may affect the activity of EGFR by palmitoylation.

In this study, using PC3 (prostate cancer) and A549 (lung cancer) cells, we explored the mechanism underlying EGFR's ligand-independent activation. We've found that FASN-dependent palmitoylation of EGFR is critical for both EGFR's ligand-independent and ligand-dependent dimerization and activation, and targeting this pathway potentiated the growth inhibitory effect of EGFR TKIs.

## RESULTS

### Ligand-independent constitutive activation of EGFR sustains the growth of cancer cells

**Figure 1:**
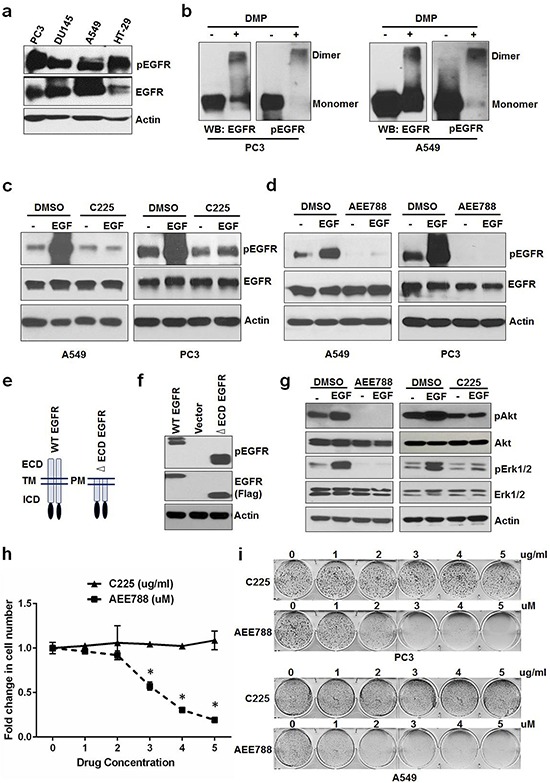
Constitutive activation of EGFR sustains cell proliferation in the absence of ligands **a.** Serum starved PC3, DU145, A549 and HT-29 cells were tested for EGFR phosphorylation. **b.** PC3 and A549 cells grown in the absence of serum/ligands were tested for EGFR dimerization (crosslinked by DMP) and phosphorylation. Serum starved A549 and PC3 were treated with EGF +/− C225 at 2.5 ug/ml **c.** or AEE788 at 2.5 uM **d.** for 15 minutes and measured for EGFR phosphorylation, EGFR and Actin using Western blot. **e.** Schematic diagram of WT and extra cellular domain deleted EGFR (ΔECD-EGFR). **f.** Western blot analysis of protein samples for pEGFR and EGFR (Flag) isolated from HEK 293 cells transfected with vector alone, WT EGFR or ΔECD EGFR for 24 hours. **g.** Western blot analysis of protein samples for pAkt, Akt, pErk1/2, Erk1/2 and EGFR isolated from PC3 cells treated with EGF +/− AEE788 or EGF +/− C225 for 15 minutes similarly as cells used in c and d. **h.** A549 cells were treated with AEE788 or C225 for 5 days at the indicated concentrations and measured cell number. **i.** Colony formation assay on PC3 and A549 cells were treated with increasing concentrations of AEE788 or C225 as indicated in 6-well plate and colony formation was counted when the cells reached 80–90% confluence. Data are means of +/− SD of triplicates (Suppl Figure 1a and 1b). Asterisk indicates the statistical significance between treated group and DMSO (*P*-value ≤ 0.0001).

Constitutive activation of EGFR in cancer cells in the absence of extracellular ligands (under serum free conditions) is well known; however, it is not clear regarding whether this activation of EGFR is sustained by extracellular or intrinsic signals. To address this question, we first examined the constitutive activity of EGFR in several cancer cell lines (PC3, DU145, A549, and HT29) cultured in serum free medium for 24 hrs. Constitutively active EGFR was detected in all of these cells (Figure 1a). We then chose two cell lines, PC3 and A549, for further investigations. Cross linking experiments revealed that EGFR constitutive activity was specifically associated with the dimerized form of EGFR (Figure 1b) in the absence of external ligands. To determine whether the EGFR constitutive activity is sustained by ligands present in the serum free medium, we added Cetuximab (C225), an antibody that blocks EGFR from binding to its ligand, into the serum free medium. As shown in Figure 1c, C225 effectively blocked EGF-induced EGFR activation but failed to inhibit the constitutive activation of EGFR. In contrast to C225, AEE788, a small molecule of EGFR tyrosine kinase inhibitor (TKI), completely blocked both the EGF-induced and the constitutive activation of EGFR (Figure 1d), suggesting that EGFR constitutive activity in the absence of serum is not mediated by extracellular ligands and might be sustained by intracellular signaling. Ligand-independent activation is well characterized for EGFR vIII, an EGFR mutant that does not bind to ligands due to the lack of part of the LBD. To further determine the role of intracellular signaling in activating EGFR, we created an EGFR mutant that lacks the entire extracellular domain (ΔECD-EGFR) and transfected it into HEK293 cells in the absence of serum. As shown in Figure 1e and 1f, both the full length EGFR and the ΔECD-EGFR could be phosphorylated, further supporting that EGFR can be activated independent of external ligands. To test the significance of this ligand-independent constitutive activity of EGFR on Akt and ERK signaling, we treated A549 with C225 or AEE788 in the absence of serum. As shown in Figure 1g, C225 blocked EGF-induced Akt and ERK phosphorylation but failed to block their basal activities, whereas AEE788 completely blocked both EGF-induced and basal activities of Akt and ERK. These results suggest that the ligand-independent constitutive activity of EGFR is required to sustain its downstream signaling pathways such as Akt and ERK. To further determine whether the ligand-independent EGFR activation is involved in sustaining cell proliferation in the absence of serum, we treated A549 cells with increasing concentration of AEE788 or C225 and measured their effects on cell growth. As shown in Figure 1h, AEE788 treatment significantly inhibited cell proliferation in a dose dependent manner, whereas C225 failed to repress cell proliferation. Consistent with the cell proliferation data, AEE788 reduced colony formation of A549 and PC3 cells in a dose dependent manner and C225 failed to show any effect on colony formation of these cells (Figure 1i and Suppl Figure 1). Together, these results suggest that ligand-independent intracellular signal dependent constitutive activation of EGFR sustains cell proliferation in the absence of external ligands.

### Constitutive activation and ligand-induced activation of EGFR are mediated by *de novo* fatty acid synthesis dependent palmitoylation

**Figure 2:**
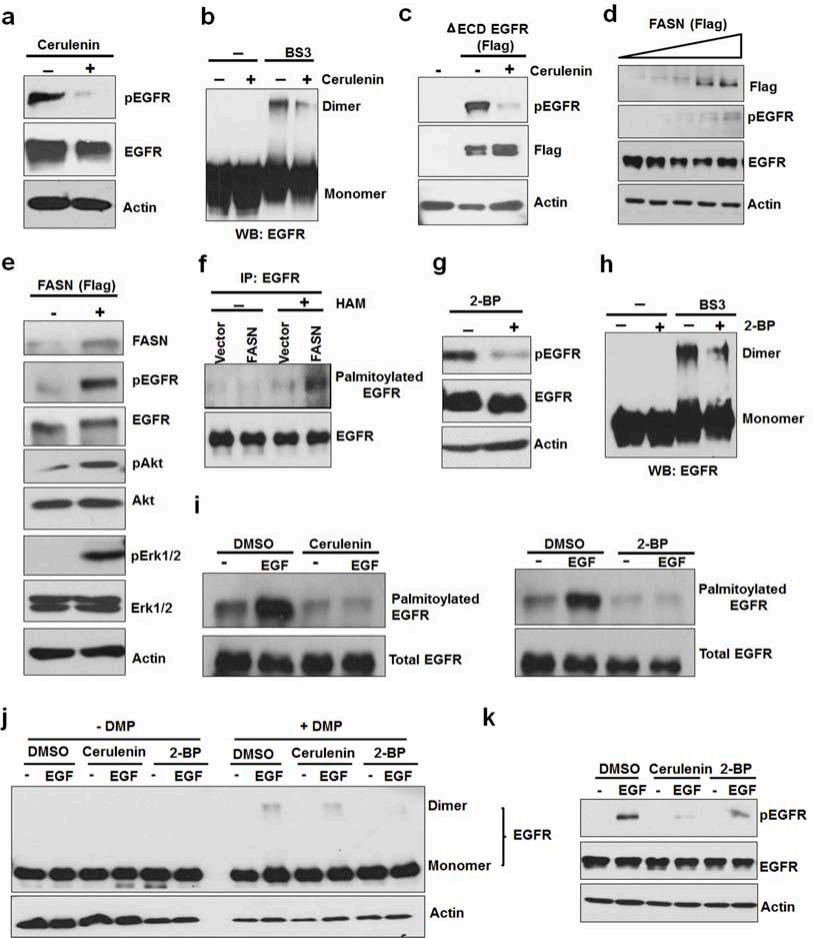
Activation of EGFR by de novo fatty acid synthesis dependent palmitoylation **a.** Western blot analysis of protein samples for pEGFR and EGFR isolated from PC3 cells treated with FASN inhibitor, cerulenin, at 5ug/ml for 8 hours. **b.** PC3 cells were treated with cerulenin for 8 hours and isolated protein samples were cross linked with BS3 as described in the materials and methods section. The cross linked samples were analyzed using Western blot for EGFR dimers. For control group, protein samples were left untreated. **c.** HEK 293 cells were transfected with ΔECD EGFR for 24 hours followed by treatment with cerulenin in serum free medium for overnight before protein isolation and Western blot analysis. **d.** Western blot analysis of protein samples for pEGFR, EGFR and Actin isolated from MCF10A cells transfected with increasing FASN (Flag tagged) concentration for 24 hours followed by serum starvation for 12 hours. **e.** Western blot analysis of protein samples (for indicated proteins) isolated from PC3 cells transfected with vector alone or FASN flag for 24 hours followed by serum starvation for 12 hours. **f.** Acyl-Biotin Exchange assay (ABE) for EGFR palmitoylation. PC3 cells were transfected with vector alone or FASN (panel 2e) and isolated samples were subjected to ABE assay for EGFR palmitoylation. HAM stands for hydroxyl amine. ABE assay was performed as described in the methods. **g.** Western blotting analysis of protein samples for pEGFR, EGFR and actin isolated from PC3 cells treated with a palmitoylation inhibitor, 2-bromopalmitate (2-BP), at 8uM for 6–8 hours. **h.** PC3 cells were treated with or without 2-BP for 8 hours and EGFR dimers were detected using cross linking agent, BS3, followed western blot analysis **i.** Western blotting of ABE assay samples for EGFR palmitoylation prepared from PC3 cells treated with DMSO or EGF (10 ng/ ml) for 20 minutes in the presence or absence of cerulenin (pretreated at 5 ug/ml for 8 hours) (left panel) or 2-BP (pretreated at 6uM for 8 hours) (right panel). **j.** EGF induced EGFR dimerization was tested in the presence of cerulenin or 2-BP (pretreated for overnight). Protein samples were prepared in the presence or absence of cross linking agent (BS3 at 3 mM) for Western blot analysis of EGFR. **k.** Western blot analysis of protein samples for pEGFR, EGFR and Actin isolated from A549 cells treated with 5ng of EGF +/− Cerulenin (5 ug/ml) or 2-BP (8 uM) for 15 minutes.

It has been shown that *de novo* fatty acid synthesis is involved in the activation of EGFR and HER2 [14, 15]. To determine whether *de novo* fatty acid synthesis is involved in the constitutive activation of EGFR, we inhibited FASN using an FASN inhibitor, cerulenin. As shown in Figure 2a, 2b, and Suppl Figure 2a, 2c, cerulenin significantly reduced the constitutive EGFR phosphorylation by reducing EGFR dimerization in the absence of serum. Furthermore, cerulenin also inhibited constitutive activation of ΔECD-EGFR expressed in HEK293 cells (Figure 2c). To further determine the role of FASN in the intrinsic activation of EGFR, we overexpressed FASN in MCF10A cells (an EGFR positive noncancerous cell line) under serum free conditions, and found that phosphorylation of EGFR was increased with the increasing levels of FASN protein (Figure 2d). Consistent with this data, over expression of FASN in PC3 (Figure 2e) and A549 cells (Suppl Figure 3) also increased the activation of EGFR and its downstream pathways (Akt and ERK) in the absence of serum. These results indicate that FASN mediated *de novo* fatty acid synthesis participates in the ligand-independent constitutive activation of EGFR.

FASN-dependent protein palmitoylation is critically involved in regulating activity, stability and cellular distribution of membranous proteins [17, 20]. The functional interrelationship between EGFR and FASN led us to speculate that palmitoylation of EGFR might be involved in EGFR activation. To test whether EGFR is palmitoylated in the absence of serum, we utilized the *in vitro* Acyl-Biotin exchange (ABE) assay to determine the palmitoylation level of EGFR immunoprecipitated from PC3 cells. As shown in Figure 2f, we found that EGFR is indeed palmitoylated, which was significantly increased by overexpression of FASN. Protein palmitoylation is catalyzed by a class or enzymes of palmitoyl acyltransferase (PAT), which can be inhibited by an inhibitor, 2-bromopalmitate (2-BP) [21]. Treatment of PC3 and A549 cells with 2-BP resulted in inhibition of constitutive activation (Figure 2g and Suppl Figure 2b) and dimerization (Figure 2h and Suppl Figure 2d) of EGFR. In the presence and absence of EGF, inhibition of FASN or PAT significantly reduced the levels of EGFR palmitoylation (Figure 2i), dimerization (Figure 2j), and phosphorylation (Figure 2k, Suppl Figure 4). Together, these data suggest that FASN mediated palmitoylation of EGFR plays a critical role in ligand-independent and ligand-dependent activation of EGFR.

### Plamitoyl acyltransferases (PATs) interact with EGFR and activate EGFR by increasing palmitoylation

**Figure 3:**
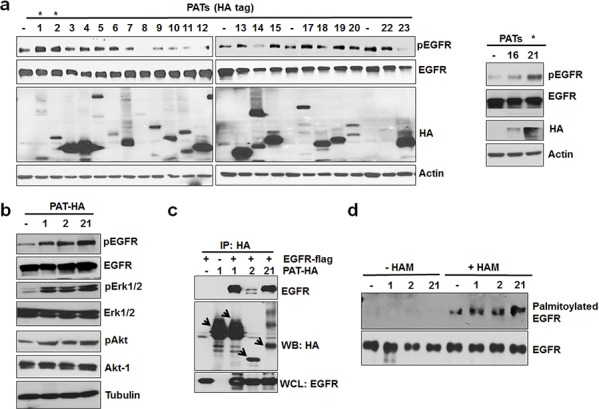
PAT 1, 2 and 21 increases EGFR activation and palmitoylation **a.** Screening of PAT enzymes for EGFR activation. A549 cells were transfected with individual PAT (HA-tagged) constructs for 24 hours followed by 12 hours of serum starvation. Isolated protein samples were tested for EGFR activation using pEGFR antibody. **b.** Western blot analysis of protein samples for pEGFR, EGFR, pAkt, Akt, pErk 1/2, Erk 1/2 and Actin isolated from A549 cells transfected with PAT plasmids 1, 2, 21 or vector alone for 24 hours followed by serum starvation for 12 hours. **c.** Western blot analysis of immunoprecipitated samples for HA and EGFR antibodies. HEK 293 cells were transfected with EGFR-flag alone or in combination with indicated PATs as shown in the Figure 3c and PAT enzymes were immunoprecipitated with HA antibody. **d.** Western blotting analysis of palmitoylated EGFR from PC3 cells transfected with vector alone or PATs.

Protein palmitoylation is catalyzed by a class of enzymes known as PATs, which transfer the palmitoyl group of palmitoyl-CoA to the SH group of cysteine residue of a target protein. There are 23 known PATs in human and rodents, and human and mouse PATs are highly homologous [22]. To further investigate the role of PATs in activating EGFR, we transiently overexpressed a panel of 23 known mouse PATs individually into A549 cells and determined their effects on EGFR activation under serum free conditions. As shown in Figure 3a, over-expression of PATs 1, 2 (>95% homologous with human PAT2), and 21 (>95% homologous with human PAT21) significantly activated EGFR, and each of these PATs also activated ERK and Akt in the absence of serum (Figure 3b). Furthermore, we also performed co-immunoprecipitation assay to test whether these PATs can physically interact with EGFR. Co-immunoprecipitation from HEK 293 cells co-transfected with flag-tagged EGFR and the HA-tagged PATs showed that PATs 1, 2 and 21 could physically interact with EGFR (Figure 3c). We also tested whether these EGFR-interacting PATs would increase EGFR palmitoylation. As shown in Figure 3d, over-expression of these PATs in A549 cells increased EGFR palmitoylation. Together, these data suggest that PATs can activate EGFR in the absence of external ligands.

### Cysteine 797 is conserved in kinase active ErbB members and is important for EGFR's palmitoylation and constitutive activation

**Figure 4:**
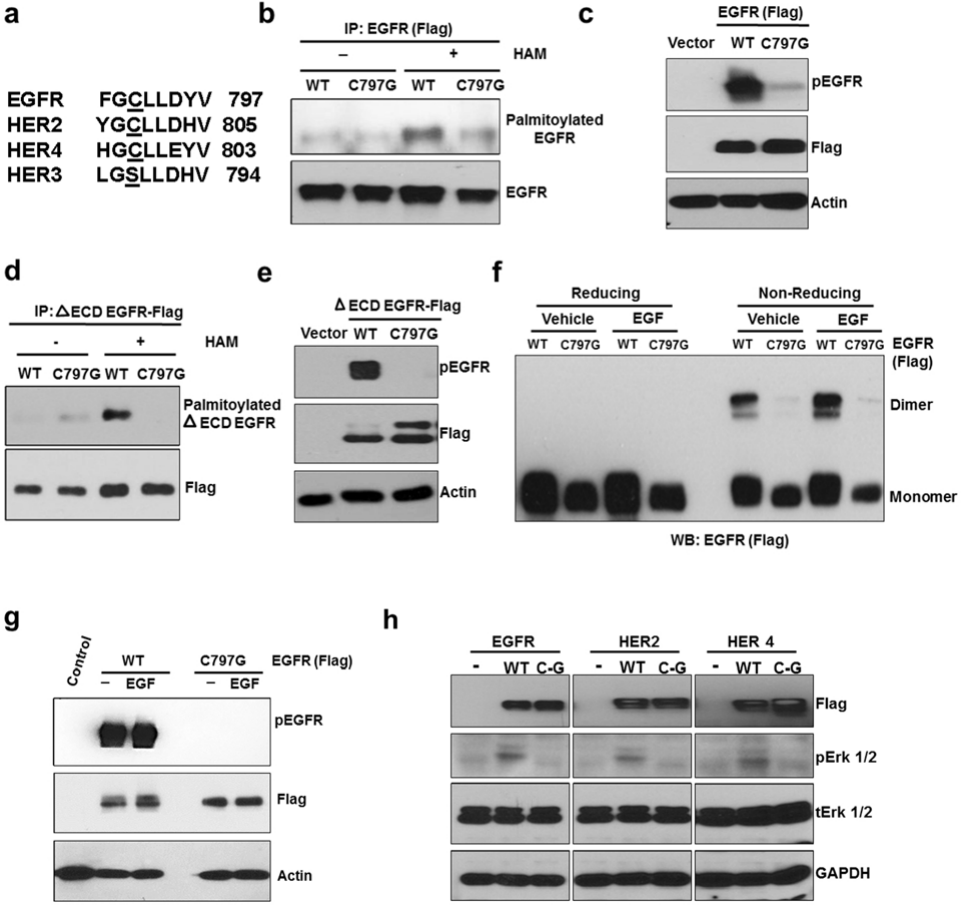
C797 site is conserved in kinase active ErbB members and is important for constitutive activation and palmitoylation of EGFR **a.** Comparison of palmitoylation site (C797) of EGFR with HER2, HER3 and HER4. **b.** Western blotting analysis for palmitoylated EGFR prepared from HEK293 cells transfected with EGFR WT or C797G mutant (Flag-tagged). **c.** C797G mutation blocks EGFR constitutive phosphorylation when expressed in HEK 293 cells. **d.** C797G mutation blocks palmitoylation of ΔECD EGFR in HEK 293 cells. **e.** C797G mutation blocks ΔECD EGFR constitutive phosphorylation in HEK 293 cells. **f.** HEK 293 cells were transfected with WT or C797G EGFR for 24 hours and treated with DMSO or EGF at 10 ng/ml for 20 minutes and protein samples were analyzed for EGFR dimers using Western blotting under reducing or non-reducing conditions. **g.** The same samples from panel f were subjected to Western blot analysis. **h.** Western blot analysis for Flag, pErk 1/2, Erk 1/2 and GAPDH. Protein samples were prepared from HEK 293 cells transfected with WT or cysteine mutants of ErbB members.

Palmitoylation of proteins is catalyzed by PATs to form thioester bond between the sulfhydryl group of cysteine residues and the carboxyl group of palmitate [23]. Program assisted prediction found that several cysteine residues in the intracellular region of EGFR could be palmitoylated (data not shown). We focused on Cysteine 797 in this study because it is conserved in all the kinase active members of EGFR family [EGFR, HER2 (Cysteine 805), and Her4 (Cysteine 803)], but not in the kinase inactive HER3 (Figure 4a). In HER3, the corresponding cysteine residue is substituted with serine. To study the role of C797 in EGFR palmitoylation and constitutive activity, we expressed wild type or C797G (Cysteine is substituted with Glycine) mutant of EGFR in HEK 293 cells. As shown in Figure 4b and 4c, C797G mutation significantly reduced palmitoylation and constitutive activity of the full length EGFR. Supportively, C797G mutation also blocked palmitoylation and activation of the ΔECD-EGFR (Figure 4d and 4e). Because dimerization is a key step for EGFR activation, we determined whether the reduced activity of C797G mutant could be due to defect in dimerization. As shown in Figure 4f, under serum free conditions, WT EGFR transfected into HEK293 cells formed dimers; however, the C797G EGFR mutant failed to form dimers regardless of EGF (Figure 4f). Consistently, EGF failed to activate the C797G mutant (Figure 4g). Furthermore, mutating the conserved cysteine sites in the other EGFR kinase-active members, HER2 and HER4, blocked their effects on activating ERK signaling in HEK293 cells (Figure 4h). These results suggest the C797 is crucial for EGFR's palmitoylation, dimerization, and activation.

### Inhibition of FASN or PATs regulates localization and protein levels of EGFR

**Figure 5:**
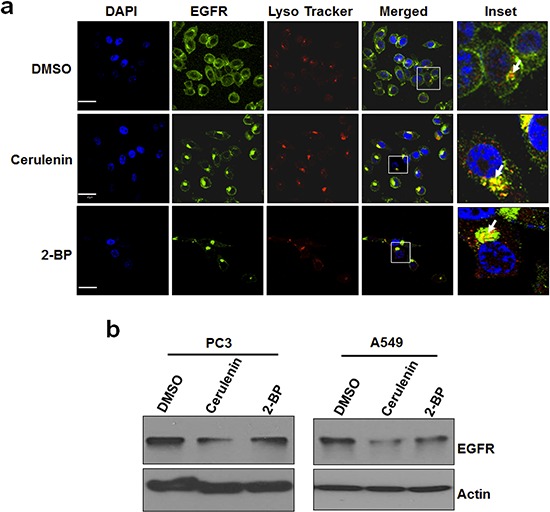
Inhibition of de novo fatty acid synthesis or palmitoylation alters EGFR cellular distribution and reduces total EGFR levels **a.** Representative immuno fluorescent images of PC3 cells. PC3 cells were treated with DMSO or cerulenin at 5 ug/ml for 24 hours and stained for EGFR (green), lysosomes (red) and nucleus (DAPI, blue). Images were taken using Olympus confocal microscope using 60x objective. Scale bar is 30um. **b.** Western blot analysis of protein samples for EGFR and Actin isolated from PC3 cells and A549 cells treated with FASN inhibitor, cerulenin at 5ug/ml or 2-BP (6 uM) for 24 hours.

Protein palmitoylation often leads to tethering proteins to the plasma membrane. To test the role of *de novo* fatty acid synthesis dependent EGFR palmitoylation in EGFR cellular localization, we treated PC3 cells with cerulenin or 2-BP, co-stained for EGFR and lysosomes, and determined EGFR localization using confocal microscope imaging. As shown in Figure 5a, EGFR is predominantly localized at the plasma membrane of the control cells, and both cerulenin and 2-BP treatment decreased EGFR levels at the plasma membrane and increased EGFR levels at lysosomes, suggesting that inhibition of this mechanism reduces plasma membranous EGFR and promotes its lysosomal translocation. Supportively, cerulenin and 2-BP also reduced EGFR protein levels (Figure 5b). These results suggest that *de novo* fatty acid synthesis dependent palmitoylation plays a critical role in maintaining the protein levels and plasma membrane localization of EGFR.

### Inhibition of FASN or PATs sensitized cancer cells to EGFR TKIs

**Figure 6:**
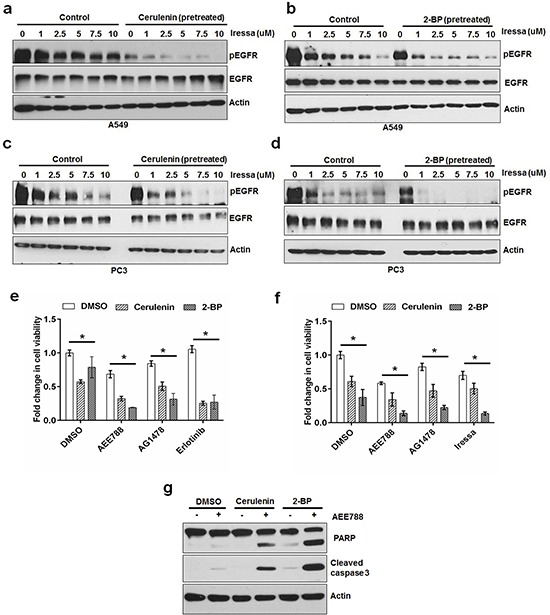
FASN or PAT inhibitor sensitizes cancer cells to EGFR TKIs Western blot analysis of protein samples for pEGFR, EGFR and Actin isolated from A549 cells treated (30 minutes) with increasing concentrations of EGFR tyrosine kinase inhibitor in the presence or absence of cerulenin at 5 ug/ml **a.** or 2-BP at 8 uM **b.** The cells were pretreated with either cerulenin or 2-BP for 5 hours before the treatment with Iressa. Western blot analysis of protein samples for pEGFR, EGFR and Actin isolated from PC3 cells treated (30 minutes) with increasing concentrations of EGFR tyrosine kinase inhibitor in the presence or absence of cerulenin at 5 ug/ml **c.** or 2-BP at 8 uM **d, e.** MTS assay in PC3 cells treated with EGFR TKI alone or in combination with cerulenin or 2-BP for 48 hours. **f.** MTS assay in A549 cells treated with EGFR TKI alone or in combination with cerulenin or 2-BP for 48 hours. Data are means of +/− SD of triplicates. Asterisk indicates the statistical significance between treated group and DMSO (*P*-value ≤ 0.001). **g.** Western blotting analysis of protein samples for PARP cleavage, cleaved caspase 3 and actin.

Knowing that EGFR's palmitoylation plays a critical role in its function, we sought to test whether inhibition of FASN or PATs activity can increase the sensitivity of cancer cells to EGFR tyrosine kinase inhibitors (EGFR TKI). We treated A549 and PC3 cells with increasing concentrations of EGFR TKI, Iressa, (1 uM to 10 uM) for 30 minutes in the presence or absence of cerulenin or 2-BP (pretreated for 3 hours). In the presence of cerulenin or 2-BP, Iressa showed an increased efficacy in inhibiting EGFR phosphorylation at every concentration compared with that of control (Figure 6a–6d). Consistently, inhibition of FASN or PATs significantly increased the sensitivity of PC3 and A549 cells to the growth inhibitory effects of EGFR kinase inhibitors (Figure 6e and 6f). In addition to the growth inhibitory effects, cerulenin and 2-BP treatment increased the pro-apoptotic effects of AEE788 as evidenced by increased levels of cleaved PARP and caspase 3 (Figure 6g). These results indicate that the *de novo* fatty acid synthesis dependent palmitoylation plays a critical role in maintenance of cancer cell survival under EGFR TKI treatment.

## DISCUSSION

EGFR is overexpressed or overactive in most cancers of epithelial origin. Based on the understanding of the canonical EGFR activation pathways, ligand induced dimerization of the receptor and activation of the tyrosine kinase activity of the receptor, anti-EGFR therapeutics have been developed for cancer therapy: monoclonal antibodies that block EGFR from binding to its activating ligands and ATP mimicking small molecule inhibitors that compete with ATP for the binding of tyrosine kinase domain of EGFR. In the clinic, the current approaches of anti-EGFR therapies have been facing critical challenges of innate resistance [5–7, 24] and inevitable acquired resistance in EGFR-positive cancers [3, 25, 26]. A better understanding on the mechanism by which EGFR executes its oncogenic functions is needed to improve the therapeutic efficacy of EGFR targeted therapies. Our understanding of EGFR functions in cancer cells has expanded in recent years, e.g. from its function at the plasma membrane to the nucleus [27] and to the mitochondrion; [15, 28, 29] from its tyrosine kinase dependent functions to its tyrosine kinase independent functions; [30, 31] from its ligand-dependent functions to its ligand-independent function [8, 9].

In this study, we have identified that ligand-independent constitutive activation of EGFR maintains the proliferative ability of cancer cells in the absence of external ligands, and *de novo* fatty acid synthesis dependent palmitoylation of EGFR is required for both the ligand-independent constitutive activation and ligand-dependent activation of EGFR. The novelty of this finding resides at the intracellular and ligand-independent nature of this EGFR activation pathway. This pathway may have potentially contributed to the resistance to anti-EGFR therapeutics in the clinic. Palmitoylation induced dimerization of EGFR may offset the effect of anti-EGFR antibody, and enhanced *de novo* fatty acid synthesis that is common in cancers and associated with chemoresistance [14, 32, 33] may counteract the growth inhibitory effect of small molecule TKIs during the intervals of TKI administration. This possibility is supported by our data that inhibition of FASN or PATs significantly potentiated TKIs’ growth inhibitory and pro-apoptotic effect.

Protein palmitoylation, the thioester linkage of palmitate moieties to cysteine residues, plays important role in regulating protein translocation, stability, and function. A wide range of proteins undergoes palmitoylation modification catalyzed by palmitoyl acyltransferases (PATs) [34]. Palmitate is, a 16-carbon saturated fatty acid, synthesized by FASN. Previously, we have found that, independent of its kinase activity, EGFR interacts with FASN at the plasma membrane in cancer cells [15]. It has been shown that expression of FASN alone is sufficient to activate EGFR and HER2 [14]. Given that plasma membrane-associated FASN plays a critical role in regulating the function of membranous proteins through protein palmitoylation, [35] the regulation of EGFR by palmitoylation was expected. Our observations that palmitoylation of EGFR is required for its dimerization and subsequent activation (Figures 2 and 4) are novel, which suggest that this mechanism plays a fundamental role in EGFR's kinase-dependent functions. The fundamentality of EGFR palmitoylation is further supported by the fact that the critical cysteine residue for EGFR palmitoylation, Cys797 of EGFR, is conserved among the kinase active members of EGFR family (EGFR, HER2, and HER4) and is substituted by a serine residue in HER3 that lacks kinase activity. The exact molecular mechanism by which *de novo* fatty acid synthesis enhances survival of cancer cells is not completely understood. Our data suggest that ligand-independent activation of EGFR by FASN-dependent protein palmitoylation might be a part of this mechanism.

Data from this study suggest that targeting EGFR's palmitoylation might be a more effective approach for EGFR based therapies, however, to achieve more specific inhibition at EGFR's palmitoylation, further studies are needed to define the molecular nature of the interplay among EGFR, FASN, and PATs.

## MATERIALS AND METHODS

### Reagents and cell lines

In this study we used two prostate cancer cell lines (PC3, DU145), lung cancer cell line (A549) colon cancer cell line (HT29), and non-cancer cell lines (MCF10A and HEK293T). All of the cancer cells were cultured in DMEM supplemented with 10% fetal bovine serum (FBS), 1% antibiotics, 5.5 mM glucose and non-cancer cells were grown in DMEM with 25 mM glucose. FASN inhibitor (Cerulenin, cat # C2389) and 2-Bromo palmitate (cat # 21604) were purchased from Sigma Aldrich. EGFR inhibitors AEE788 (Cat# S1486), Iressa (Cat #S1025), Erlotinib (Cat #S7786) and AG1478 (Cat # S2728) were purchased from Selleck Bio. Antibodies for FASN (cat # sc-55580), EGFR (Cat # sc-03), beta actin (Cat # sc-1616) and Tubulin (Cat # sc-5286) were purchased from Santa Cruz Biotechnology. EGFR monoclonal antibody, C225 (Cat #MABF120), was from EMD Millipore for EGFR immuno precipitation studies. Anti-EGFR for immuno staining (Cat #4267), Akt, pAkt, Erk1/2 (cat #9102) and pErk1/2 (Cat #9101) were obtained from Cell Signaling. Anti-flag (M2) antibody (Cat # F3165) was from Sigma Aldrich. Anti-pEGFR (Y1137) (Cat # 44794G) antibody was from Invitrogen. Lysotracker Red (Cat #L-7528) was purchased from life technologies.

### Cloning and mutagenesis

EGFR, HER2 and HER4 were PCR amplified from PCDNA3.1 constructs and sub cloned into pRK5F (flag-tag) vector. Specific cysteine mutations on EGFR constructs were created using Stratagene Quickchange Lightening site directed mutagenesis kit purchased from Agilent technologies (Santa Clara, CA). Primers for EGFR C797G mutation are “CGCAGCTCATGCCCTTCGGCGGCCTCCTGGAC TATGTCCGG (Forward) and CCGGACATAGTCCAGGAGGCCGCCGAAGGGCA TGAGCTGCG (reverse). Primers for HER2 C805G mutation are “GCTTATGCCCTATGGCAGCCTCTTAGACCATG TCCGG” (forward) and CCGGACATGGTCTAAGAGGCTGCCATAGGGCA TAAGC (reverse). Primers for HER4 C803G are CTTATGCCCCATGGCGGCCTGTTGGAGTATGTCC (forward) and GGACATACTCCAACAGGCCGCCATGGGGCATAAG (reverse). The HA-tagged PAT expression plasmids [36] were obtained from Dr. Masaki Fukata at the National Institutes of Natural Sciences, Okazaki, Japan, as a gift.

### Transfection and western blotting

Plasmid transfections were performed using lipofectamine 2000 according to the manufacturer instructions. Protein samples were prepared by lysing the cells in RIPA buffer (Cat # R0278, Sigma Aldrich) supplemented with protease inhibitors and phosphatase inhibitors on ice for 30 minutes. Lysed cell lysates were collected and centrifuged at 14000 rpm for 15 minutes at 4°C. Equal amount of proteins were subjected to the SDS PAGE and western blotting analysis for proteins of interest using antibodies at optimized concentrations.

### Immunoprecipitation and immunostaining

Equal amount (500 ug) of protein samples were added to the eppendorf tube containing 25ul of protein A/G beads and 1ug of antibody and incubated at 4°C for overnight on a shaker. After overnight incubation, the beads were washed with 600ul of RIPA buffer for three times. Immunoprecipitated proteins were eluted directly into lamellae buffer and boiled for 5 minutes and subjected to western blotting. Immunocytochemistry (ICC) was performed as described by previously.

### EGFR dimerization assay

EGFR dimers were detected through western blotting analysis using non-reducing conditions (Bollu et al 2014) and cross linking agents (BS3 and DMP). After treatments with drugs, cells were washed with PBS and incubated with 3 mM BS3 (Cat # 21580, Thermoscientific) or DMP (Cat # D8388, Sigma Aldrich) at 12 mg/ml for one hour on shaker at 4C. Reaction was quenched by adding 250 mM glycine (for BS3 method) or 50 mM ethanolamine (for DMP method) for 5 minutes at 4C. Cells were washed with ice cold PBS and lysed with RIPA buffer.

### *In vitro* palmitoylation assay

The Acyl-Biotin exchange palmitoylation assay was carried out according the protocol used by others. EGFR was immuno precipitated from protein samples using 1 ug of EGFR monoclonal antibody, C225. To block non-palmitoylated cysteine residues, immuno precipitated EGFR was treated with 50 mM NEM in RIPA buffer at 4°C for 2 hours on a shaker followed by three washes with RIPA buffer. EGFR was then treated with hydroxylamine (HAM) buffer to remove palmitate from Cysteine residues (1 M Hydroxylamine, 50 mM tris, 150 mM Nacl, 5 mM EDTA, 0.2% TX100, pH 7.4) at room temperature for 2 hrs on a shaker. For mock, EGFR was treated with same buffer without hydroxyl amine. EGFR was then treated with 4 uM of BMCC-Biotin in 50 mM tris, 150 mM Nacl, 5 mM EDTA, 0.2% TX100, pH 6.2 for 1–2 hours followed by three washes to remove excess biotin. 60 ul of reducing protein sample buffer was added and samples were boiled for two minutes to elute EGFR from beads. 20% of sample was loaded on to SDS PAGE gel. Membrane was blocked with 5% BSA for overnight followed by incubation with streptavidin conjugated with HRP at 1:30000 for 60 minutes and Biotin-streptavidin HRP complexes were visualized by exposing the membrane to ECL and then to X-ray film.

### Cell proliferation assay

Seven day cell proliferation assays were carried out in 96-well plates by measuring the cellular DNA content using a FluoReporter Blue fluorometric double-stranded DNA Quantitation kit (Life Technologies cat#: f-2962) following the manufacturer's protocol. Florescence was measured at 360 nm excitation and 460 nm emission.

### Colony formation assay

1000–2000 cells were plated in each well of 6-well plates and grown in DMEM supplemented with 5% charcoal stripped serum for 24 hours followed by treatment with indicated concentrations of drugs. Medium was changed every 72 hours until the control cells reached 80–90% confluence. At the end of the experiment, the cells were fixed with 4% PFA and stained with 2.5% crystal violet for 10 minutes. Images were taken using a scanner.

### MTS assay

Cell viability assay was performed using a MTS assay kit (Cat # G3582, Promega) following manufacturer instructions in 96 well plates.

### Statistics

Student two-tailed *t*-test was used to compare the values (mean ± SD) of triplicate control and experimental groups of three independent experiments. *P* < 0.05 is considered as significant difference.

## SUPPLEMENTARY FIGURES


