# Supplementary material for: Intracellular activation of EGFR by fatty acid synthase dependent palmitoylation
Source: Oncotarget. 2015 Sep 12;6(33):34992–5003. doi: 10.18632/oncotarget.5252 (PMC4741504; doi:10.18632/oncotarget.5252)
Supplement: Supplementary file 1 [file oncotarget-06-34992-s001.pdf]

SUPPLEMENTARY FIGURES

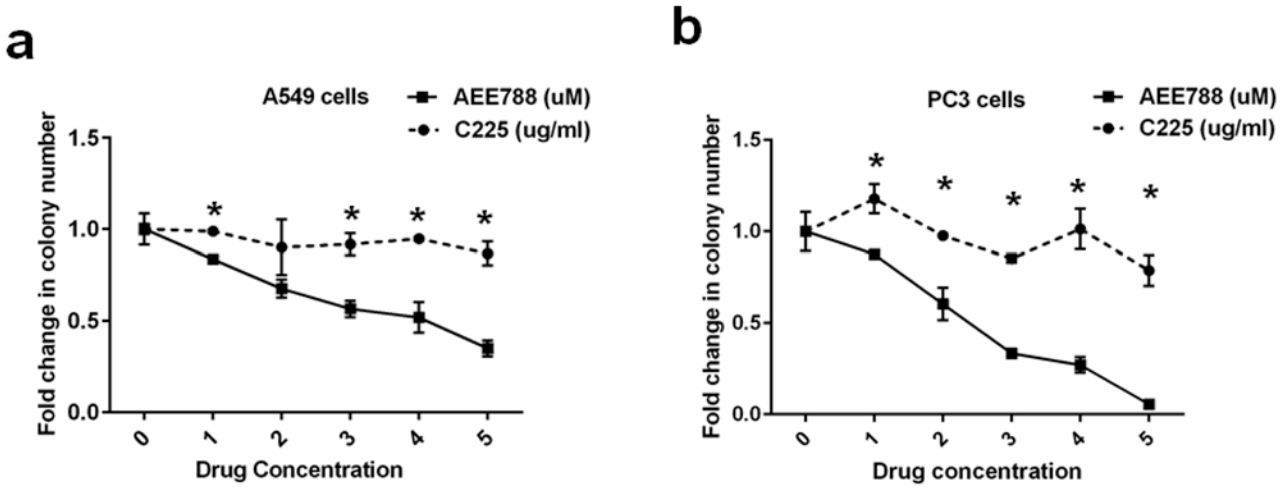

Supplementary Figure S1: Quantification of colony formation shown in Figure 1i. A549 **a.** and PC3 cells **b.** treated with increasing concentrations of AEE788 (uM) or C225 (ug/ml) and colonies were counted manually. Data are means of +/− SD of triplicates. Asterisk indicates the statistical significance between treated group and DMSO (*P*-value ≤ 0.01).

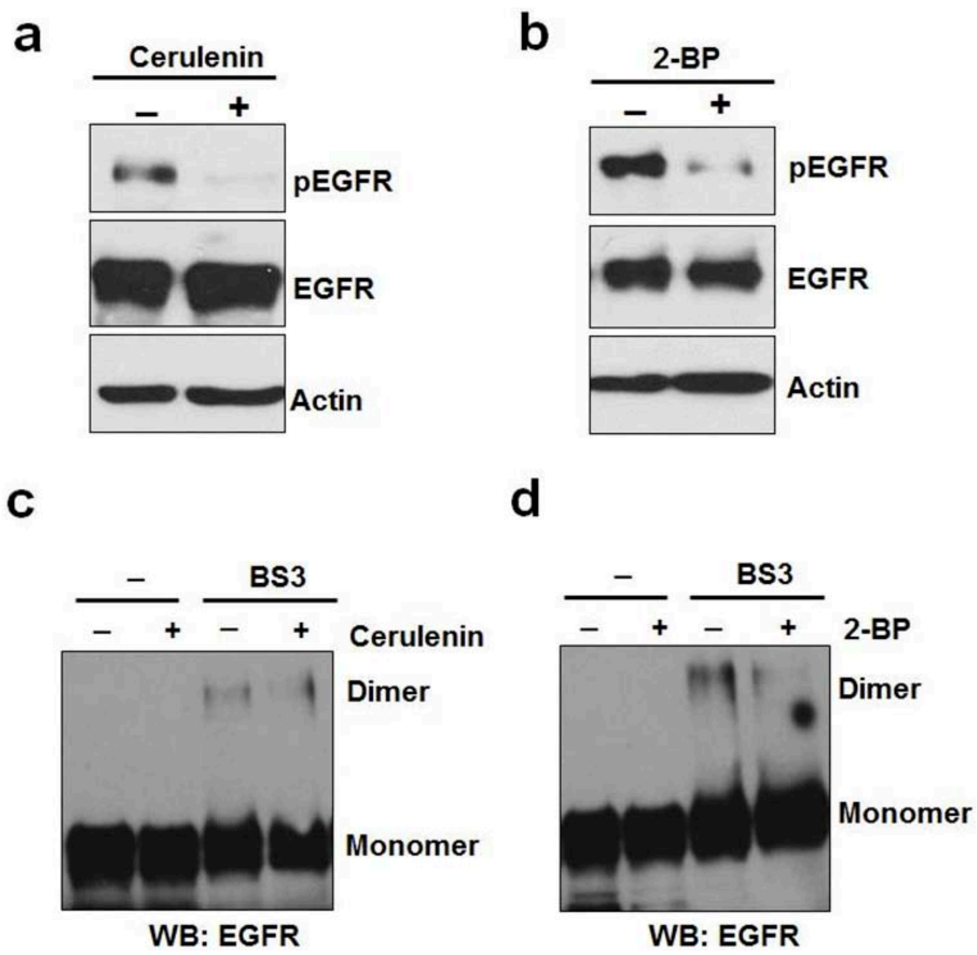

Supplementary Figure S2: FASN and PAT inhibitors reduce EGFR phosphorylation and dimerization in A549 cells. Serum starved A549 cells were treated with cerulenin at 5 ug/ml or 2-BP at 6 uM for 8 hours and tested for EGFR phosphorylation **a.** and **b.** and dimerization **c.** and **d.** using crosslinking agent (BS3) and Western blot analysis.

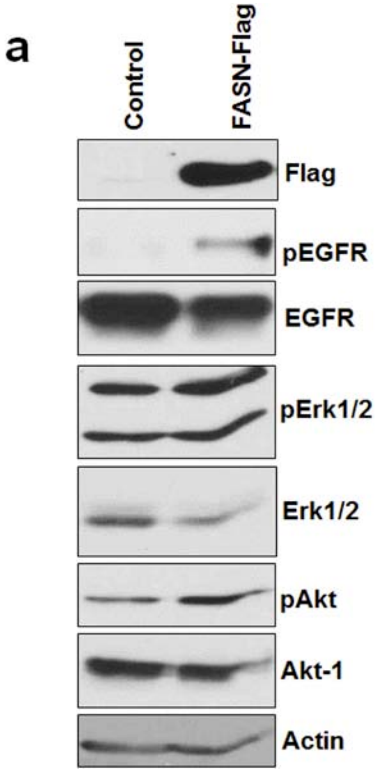

Supplementary Figure S3: Expression of FASN increases EGFR signaling in A549 cells. Protein samples were isolated from PC3 cells transfected with vector alone or FASN (Flag-tagged) for 24 hours followed by serum starvation for 12 hours and Western blot analysis was carried out for Flag tag, pEGFR, pAkt, Akt, pErk 1/2, Erk1/2 and Actin.

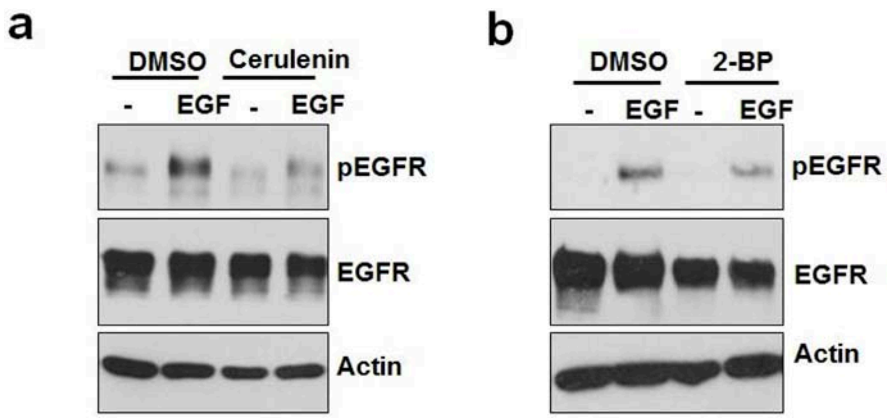

Supplementary Figure S4: FASN and PAT inhibitors reduce EGF induced EGFR phosphorylation in PC3 cells. Serum starved PC3 cells were treated with cerulenin at 5 ug/ml **a.** or 2-BP **b.** at 6 uM for overnight and treated with EGF as indicated for 15 minutes. Isolated protein samples were subjected to Western blot analysis for pEGFR, EGFR and actin.
